# Congenital heart disease in a patient with COVID-19 infection during early pregnancy: a case report

**DOI:** 10.1186/s43044-022-00295-5

**Published:** 2022-08-27

**Authors:** Mohamed Ali Abdelkader, Hamed Mohamed Abbas, Ibrahim Mohamed Aboelkhair, Aliaa Salah Ali Alafify, Basim Abdelfattah Elgazzar, Mai Salah El-Din Koura

**Affiliations:** 1 Fetal Medicine Unit, Cairo University, Cairo, Egypt; 2 Menoufia University, El-Menoufia, Egypt; 3 Magdi Yacoub Heart Foundation, Aswan, Egypt; 4 Faculty of Medicine, Menoufia University, El-Menoufia, Egypt

**Keywords:** COVID-19, Pregnancy, Congenital heart disease, Case report

## Abstract

**Background:**

Since the end of 2019, the world has been afflicted by a coronavirus pandemic caused by coronavirus 2 (severe acute respiratory syndrome) (SARS-CoV-2). COVID-19 causes a wide range of signs and symptoms with varying consequences. The impact of the COVID-19 infection on pregnant women and their fetuses is still under investigation.

**Case presentation:**

A case of a 34-years-old non-vaccinated pregnant woman who had a COVID-19 infection in the first month of her pregnancy and went into premature labor at 34 weeks was reported. Congenital heart disease and hydrops were present in the fetus. The infant girl was cyanotic after delivery, experienced bradycardia, and was in poor overall condition; she was admitted to the NICU and died 5 days later.

**Conclusions:**

Some theories suggest that SARS-CoV-2 may be transmitted vertically from mother to fetus. Congenital abnormalities can be caused by a variety of viruses. Although, congenital heart diseases can occur due to different causes, we suggest that COVID-19 may play a role in the development of congenital heart defects.

## Background

COVID-19 is one of the coronaviruses that can cause a wide range of symptoms and results. The disease’s effects on pregnant women and their fetuses are still being researched. Because of functional alterations in early embryonic cells, viral infections during the beginning of pregnancy can induce congenital defects in the newborn [1, 2]. Viruses such as varicella and rubella can cross the placenta and harm the fetus [2]. Miscarriage, growth restriction, hydropsis, and even death are all possible outcomes of viral infection in fetuses [2]. Infection with the SARS-CoV virus in 2002/2003 resulted in a significant number of abortions and premature births [2]. In the first 6 gestational weeks, SARS-CoV-2 has a higher chance of developing congenital birth defects [1].

## Case presentation

**Fig. 1:**
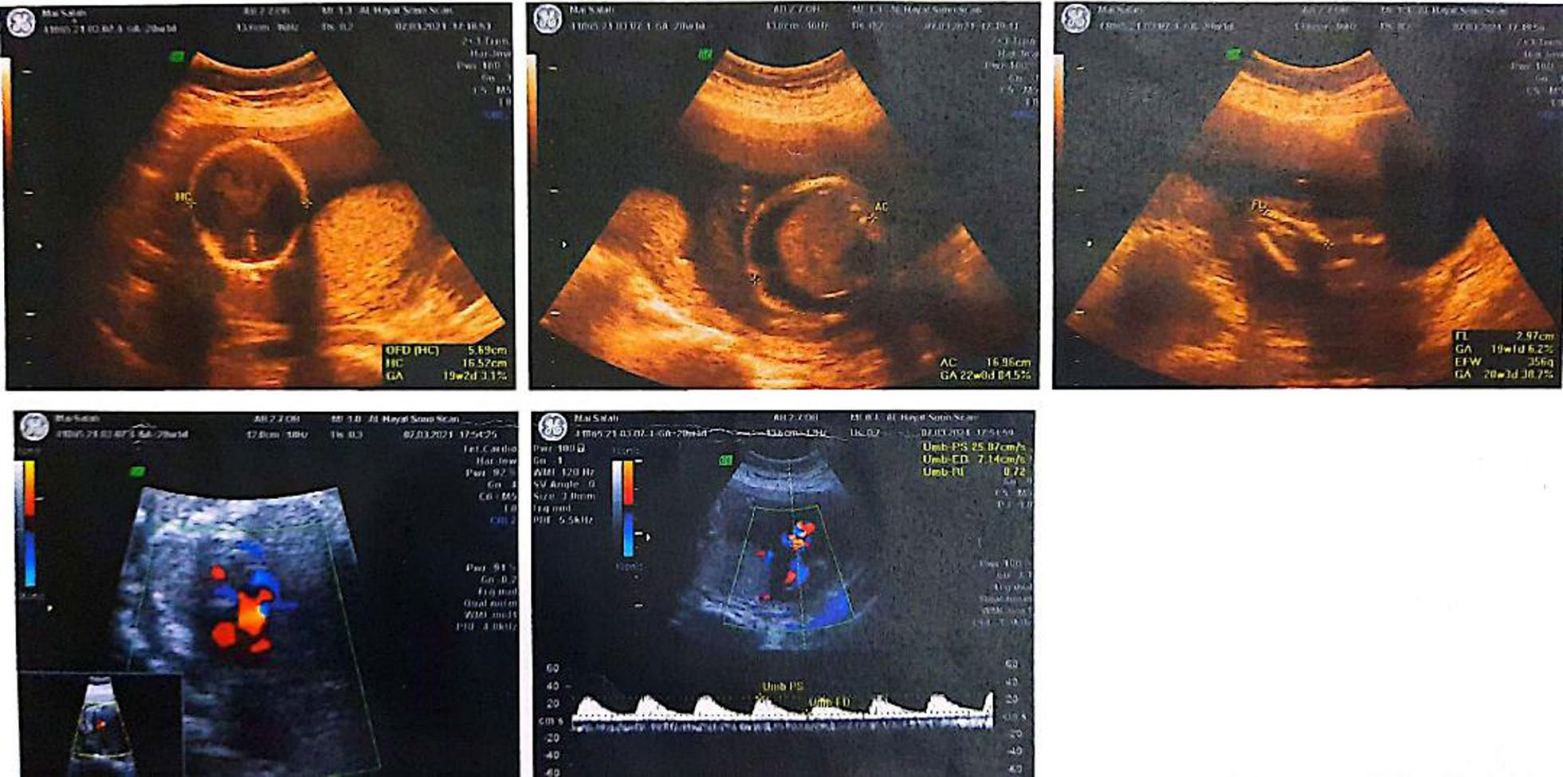
4D obstetric ultrasonography in the 20th gestational week

This presentation details the case of a 34-years-old non-vaccinated pregnant woman who had a COVID-19 infection during the first month of pregnancy. She was given azithromycin and acetaminophen, both of which have been shown to be safe in terms of causing preterm birth and birth abnormalities [3]. There were no congenital birth defects in the family. In the 17th week of pregnancy, fetal ascites was identified. In this week's 4D obstetric ultrasonography, substantial fetal ascites was seen, as well as an abnormal four-chamber image of the fetal heart with a major atrial septal defect (ASD). The fetal heart showed an atrioventricular valve (AV) canal defect during the second 4D obstetric ultrasonography in the 20th gestational week. There was a significant amount of fetal ascites (Fig. 1).

**Fig. 2:**
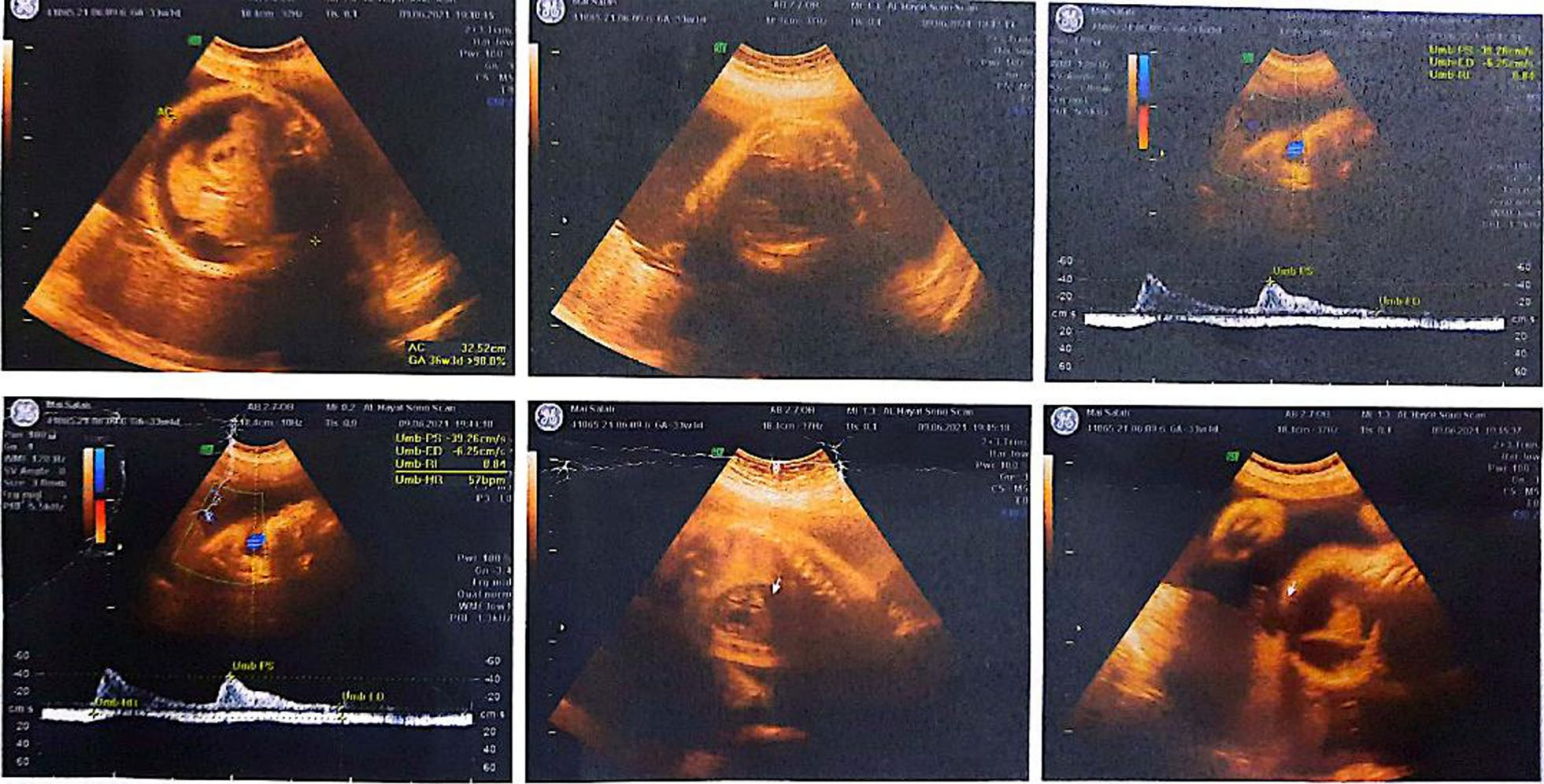
4D obstetric ultrasonography in the 34th gestational week

In the 34th week of pregnancy, a 4D obstetric ultrasonography revealed an AV canal defect and fetal hydrops (marked ascites, pericardial effusion, and skin edema) Fig. 2. Fetal bradycardia (48–56 beats per minute) was detected.

Polyhydramnios was also noticed. The mother had been edematous and had hypoalbuminemia (2.6 g/dL) over the past week.

A preterm cesarean delivery took place on June 13, 2021. The umbilical cord and placenta were normal. The infant girl was 34 weeks old, the third sibling of a negative consanguineous marriage with no maternal history of any risk factor, and her weight was 2.5 kg, according to the pediatric checkup performed after birth. She exhibited cyanosis, AV canal defect, bilateral choanal atresia, hydrops fetalis, and bradycardia (60 beats per minute) and was in poor general condition. The infant girl was admitted to the neonatal intensive care unit (NICU), where she was intubated and placed on mechanical ventilation (MV). She took amikin, atropine, and ampicillin/sulactam as a pharmacological treatment in the NICU.

An echocardiogram was performed on the infant girl on the 1st and 3rd days after birth, revealing only two pulmonary veins were seen connected to the left atrium, left-sided superior vena cava, and common atrium due to large secundum ASD and a common AV valve with mild regurge on the right side of the valve. There was also severe regurge on the left side, an inlet ventricular septal defect (VSD) with large perimembranous extension and malposed great arteries with the aorta to the right and overriding the VSD, and the pulmonary artery to the left with pulmonary atresia and hypoplastic confluent pulmonary arteries with no antegrade flow. Trivial aortic regurgitation (AR), a left-sided arch with a slender descending aorta and pulsatile flow, was also seen. Pericardium was normal.

General ultrasonographic assessment and blood tests for the infant girl were performed on the 4th day of her life. She experienced germinal matrix hemorrhage (grade 1), brain edema, and hypoxic-ischemic encephalopathy (grade 3), according to the results of the cerebral vascular investigation. According to the abdominal and pelvic exams, the liver was left-sided and the spleen was right-sided. There have also been reports of significant ascites with intact abdominal organs.

A normal karyotype was found in the infant girl. Hypoalbuminemia (2.2 g/dl), leukocytosis (20.9 × 10^9^/L), thrombocytopenia (105 × 10^9^/L), and a normal hemoglobin serum level (12.9 g/dl) were all present in her blood. The infant girl died on her 5th day of life. On the 10th day after giving birth, the mother's albumin level returns to normal (4.6 g/dL).

## Discussion

Preterm labor (before 34 or 37 gestational weeks) was the most common COVID-19 side effect in pregnant women [2, 4]. Preeclampsia-related neonatal mortality, preterm membrane rupture, and fetal development restriction were among the others [2, 4]. Fetal distress, neonatal asphyxia, a high rate of NICU admission, stillbirth, and neonatal death were the most common outcomes reported in fetuses and neonates [4].

In some research, vertical transmission of coronavirus (SARS-CoV-2) from mother to fetus has been suggested [1, 2, 4]. A newborn infant born to a woman infected with SARS-CoV-2 had higher IgM antibody and cytokine levels 2 h after birth, implying in utero viral infection because IgM antibodies cannot cross the placenta [5].

The virus can pass the blood–brain barrier and influence the neurological system, as evidenced by the presence of viral IgM in cerebrospinal fluid [1, 2].

In another case, the placenta of a stillborn fetus tested positive for SARS-CoV-2, and the umbilical cord connective tissue was inflamed, indicating fetal inflammatory response [2]. In another example of SARS-CoV-2 infection in a pregnant woman, the placenta contained inflammatory infiltrates, indicating infection transfer from mothers to their fetuses [2].

The virus needs angiotensin-converting enzyme 2 (ACE2) and S protein protease receptors to enter the cell, and these receptors are found in developing human embryos in the early stages of development (gametes, zygotes, and 4-cell embryos) [1, 2]. As a result, the virus has the ability to penetrate fetal cells at an early stage of development and affect cell transformation and growth, a theory that tries to illustrate the mechanism of SARS-CoV-2 transmission from mother to fetus in early pregnancy [1, 2].

The human heart is one of the earliest organs to develop and begin functioning during the embryonic period. At week 7 of pregnancy, the four-chambered heart is fully formed [6]. The failure of the endocardial cushions to fuse during the embryonic development of the heart causes an atrioventricular septal defect (AVSD) [7].

A meta-analysis found that when compared to mothers who did not have a viral infection at the start of pregnancy, a viral illness such as rubella and cytomegalovirus infections in early pregnancy greatly increases the chance of developing heart diseases in offspring [8].

The abnormal accumulation of fluid in two or more fetal compartments, such as the abdominal cavity, pleural effusion, pericardial effusion, and skin edema, is referred to as hydrops fetalis. One of the reasons for fetal hydrops is viral infection [2]. SARS-CoV-2 infection was recently reported in two pregnant women, and their fetuses had fetal transient cutaneous edema [9]. One of the most common causes of fetal hydrops is congenital heart abnormalities [10].

The absence of additional risk factors that can cause complete AVSD other than covid-19 infection at the beginning of the pregnancy led us to suggest that Covid-19 may be linked to congenital heart disease (CHD) in this particular case. The biggest risk factors for complete AVSD, according to Myoclinic medical database, are Down syndrome, rubella or other viral infections, diabetes mellitus, smoking, alcohol, and specific drugs [11].

As we previously indicated, the mother in question had no history of illness, was not diabetic or hypertensive, did not smoke or drink, and was not taking any medications prior to or throughout the first trimester of pregnancy. The mother was 34 years old; prior research has shown that neither pregnancy outcomes nor risk factors for fetal congenital abnormalities are affected by this age [12, 13].

One of the study's limitations is the lack of fetal autopsy and placental histological examination, but based on the aforementioned findings and in line with recent case reports, we suggest that covid-19 infection of the mother at beginning of pregnancy might play a role in producing this rare and complicated CHD in the case that was accompanied by encephalopathy and hydrops fetalis.

The American Heart Association (AHA) mentioned in its guidelines for the diagnosis and management of fetal cardiac illness that prenatal virus exposure may be linked to positive cardiac findings when ultrasonography signs such as effusions or hydrops are present [14]. The infant girl in our case showed hydrops fetalis, which may imply CHD caused by a viral infection. Although, hydrops may be a result of CHD [10].

Because polyhydramnios is highly correlated with fetal AVSD, echocardiograms should be performed when polyhydramnios is detected by ultrasound in any mother who has CHD risk factors, such as viral infection [15].

Numerous recent investigations illustrate that the SARS-CoV-2 virus can pass through the placenta and affect the fetus [16, 17], which is based on immunohistochemical tests and the confirmation of non-immunologic hydrops fetalis.

Finally, an editorial opinion report about two case reports that presented three neonates without any positive reverse transcriptase-polymerase chain reaction test results to any infant specimen gave the conclusion that they had been exposed to SARS-CoV-2 in utero from their mothers based on elevated IgM and IgG antibody values in the neonates' postnatal blood and recommended further investigations [18].

Although, all case reports discussed the effects of covid-19 on fetuses in the second and third trimesters, results are in line with our suggestion that SARS-CoV-2 can affect fetuses. Further observational studies should be done to investigate covid-19 effect on mothers and fetuses at early pregnancy.

## Conclusions

In conclusion, SARS-CoV-2 can have serious consequences for both pregnant women and their fetuses. As a result, it is critical to investigate the impact of SARS-CoV-2 infection on fetuses at an early stage of pregnancy in order to handle any fetal defects that may occur and decrease neonatal morbidity and mortality. In this case, we suggested that there might be a relationship between covid-19 mother infection at the beginning of pregnancy and congenital heart disease of the fetus. This suggestion needs further investigations and more observational studies to obtain accurate results about the correlation between covid-19 mother infection at early pregnancy and congenital defects.

## Data Availability

The datasets used during the current study are available from the corresponding author on reasonable request.

## Abbreviations

SARS-CoV-2: Severe acute respiratory syndrome coronavirus 2
ASD: Atrial septal defect
AV: Atrioventricular valves
NICU: Neonates’ intensive care unit
MV: Mechanical ventilation
VSD: Ventricular septal defect
AR: Aortic regurgitation
ACE2: Angiotensin-converting enzyme 2
AVSD: Atrioventricular septal defect
CHD: Congenital heart disease
AHA: American Heart Association
